# Biodegradable mulch films promote sugarcane growth by regulating rhizosphere microbial communities and predicted nutrient cycling-related functional potentials

**DOI:** 10.3389/fmicb.2026.1918463

**Published:** 2026-08-26

**Authors:** Qianyuan Duan, Qiang Liu, Xinping Mao, Zhaonian Yuan, Ziqin Pang, Yufang Shen

**Affiliations:** 1 State Key Laboratory of Soil and Water Conservation and Desertification Control, Northwest A&F University, Yangling, China; 2 College of Natural Resources and Environment, Northwest A&F University, Yangling, China; 3 Institute of Agricultural Resources and Environment, Ningxia Academy of Agro-forestry Science, Yinchuan, China; 4 National Engineering Research Center for Sugarcane, Fujian Agriculture and Forestry University, Fuzhou, China; 5 Xianghu Laboratory, Hangzhou, China

**Keywords:** biodegradable mulch film, fungal-bacterial co-occurrence network, microbial network characteristics, soil properties, sugarcane yield

## Abstract

**Introduction:**

Conventional polyethylene (PE) mulch causes persistent soil plastic pollution; biodegradable mulch films (BMFs) may avoid this, but their effects on sugarcane rhizosphere microbes and yield are unclear.

**Methods:**

Using a field trial in Yunnan, we compared polyethylene mulch (PM) with two PBAT/PLA-based biodegradable mulch films (BMFs) differing in thickness (Thick Film, BTK, 0.008 mm; Thin Film, BTH, 0.005 mm). Rhizosphere soil (0–40 cm) physicochemical properties, bacterial (16S) and fungal (18S) communities, predicted microbial functional potentials, and sugarcane agronomic traits were analyzed.

**Results:**

BMFs degradation released organic carbon that significantly altered the rhizosphere microenvironment, increasing soil pH, organic matter, and available potassium relative to PM. These physicochemical changes drove microbial community differentiation. Compared with PM, BMFs increased the relative abundances of *Chloroflexi, Acidobacteria*, and *Basidiomycota*, while decreasing *Actinobacteria* and *Ascomycota* (*p* < 0.05). BMFs also enhanced microbial α-diversity, with bacterial diversity being higher under BTK and fungal diversity under BTH. Neutral model analysis revealed that bacterial community assembly was dominated by stochastic processes; however, BMFs reduced dispersal limitation and strengthened deterministic environmental filtering, particularly under the BTH treatment, due to enhanced microhabitat heterogeneity from faster degradation. These community shifts were associated with enriched predicted sulfur (dsrAB) and nitrogen (nosZ, nif) cycling genes and more complex, cooperative bacterial–fungal interaction networks, with BTH exhibiting higher network complexity and positive interactions. These microbial responses were associated with improved sugarcane performance. Compared with PM, BMFs increased single stalk weight by 8.76%−11.68%, millable stalk number by 4.99%−7.38%, and cane yield by 17.11%−17.40%.

**Discussion:**

Collectively, our results demonstrate that BMFs, through degradation-driven microhabitat alteration, strengthen deterministic assembly, promote functionally specialized taxa and cooperative networks, and enhance predicted nutrient cycling potentials, thereby improving sugarcane productivity and offering a sustainable alternative to PE mulch.

## 1 Introduction

Plastic film mulching is a widely adopted agronomic practice worldwide (Li et al., 2024; Hu W. et al., 2025), particularly in arid and semi-arid regions (Ren et al., 2023; Zhang et al., 2024a), because it offers substantial advantages in optimizing soil moisture and temperature conditions (Niu et al., 2020; Zhao et al., 2020), suppressing weed growth (Khalid et al., 2023), and ultimately enhancing crop yield and water use efficiency (Zhang X. et al., 2022; Zhang W. et al., 2025). It has been reported that conventional polyethylene (PE) film mulching contributes nearly half of the yield increase in dryland agroecosystems (Liu et al., 2025). In China, the mulched area covered by plastic films has reached approximately 50 million hectares, substantially enhancing national food security (Wang K. et al., 2025). Sugarcane (*Saccharum officinarum L*.) is a typical high-yield sugar crop and an important biofuel feedstock in tropical and subtropical regions of China and worldwide (Lam et al., 2009; Wu et al., 2025). As a high-biomass crop, sugarcane has high water requirements, and water deficits can markedly reduce yield, particularly during the grand growth stage, which is highly sensitive to water stress (Dingre and Gorantiwar, 2021). In addition, the seedling stage requires stringent hydrothermal conditions. Meanwhile, weed infestation can also significantly reduce sugarcane yield (Li and Yang, 2015). In response to these growth characteristics, plastic film mulching has been widely applied in local sugarcane cultivation systems. This practice increases early-season soil temperature and maintains surface soil moisture, thereby meeting critical hydrothermal requirements during the seedling stage (Qin et al., 2014). At the same time, it suppresses weeds through light exclusion and reduces pest pressure and resource competition (Yuan et al., 2025), effectively promoting crop emergence and early growth (Zhao et al., 2023). These effects mitigate the sensitivity of sugarcane to water and temperature fluctuations throughout its growth cycle, thereby enhancing production stability and resource use efficiency in regional sugarcane agroecosystems while reducing production costs.

However, although PE film mulching has substantially increased agricultural productivity, it has also resulted in severe “white pollution” in farmland soils (Yin et al., 2019; Meng et al., 2022). Due to their low degradability, PE mulch films leave large quantities of plastic fragments that persist in soils for extended periods following tillage practices, wind and water erosion, and biochemical processes (Liang et al., 2024). Residual plastic film fragments accumulate in soils and are recognized as a major source of microplastics (MPs, diameter < 5 mm) (Qiu et al., 2024). These MPs can migrate from soils to aquatic environments through gravitational settling and precipitation-driven leaching (Jiao et al., 2022). Within soils, accumulated MPs disrupt soil structure, reduce porosity and permeability (Hu Q. et al., 2023), decrease nutrient storage capacity (Meng et al., 2022), and alter the diversity and evenness of rhizosphere microbial communities (Han et al., 2025; Wang H. et al., 2025). In addition, MPs exhibit a strong capacity to adsorb heavy metals, and their co-occurrence in soils can exert synergistic toxic effects on soil biota (Zhang K. et al., 2025), thereby threatening the sustainability of agroecosystems.

To address the environmental risks associated with PE mulch films, BMFs have been proposed as a promising alternative (Huang F. et al., 2023). These materials are typically composed of natural or synthetic polymers, such as polylactic acid (PLA), polybutylene adipate/terephthalate (PBAT), or starch-based polymers (Parida et al., 2024). Through photo-oxidation and biodegradation, they gradually fragment into low-molecular-weight organic compounds under the combined effects of light, temperature, and microbial activity (Fan R. et al., 2024; Li et al., 2025), and are ultimately mineralized into CO_2_ and H_2_O (Sintim et al., 2019; Liang et al., 2025). Several studies have demonstrated that, compared with conventional PE and polypropylene (PP) films, BMFs not only effectively mitigate the risk of plastic residues but also improve crop growth environments across multiple dimensions of soil physical, chemical, and biological properties, ultimately enhancing crop productivity. In terms of soil physical properties, BMFs reduce soil bulk density and increase aggregate stability and water infiltration capacity (Sintim et al., 2021). With respect to soil biological and chemical regulation, BMFs enhance the activities of soil enzymes such as urease and catalase, alter microbial community composition, and increase microbial activity and network complexity (Bai et al., 2025; Liang et al., 2025). Meanwhile, BMFs enhance carbon sequestration by increasing soil organic matter content, regulating carbon fractions within aggregates of different size classes, and reducing soil organic carbon (SOC) mineralization rates (Wang K. et al., 2024). In addition, BMFs improve the availability of soil nutrients, including nitrogen and phosphorus, thereby further enhancing soil fertility (Iamsaard et al., 2025). Furthermore, BMFs significantly reduce plastic pollution and residual film accumulation in farmland soils, lowering the risks of soil fertility degradation and environmental contamination (Xiong et al., 2024). Building upon these improvements in soil conditions, BMFs enhance water use efficiency by optimizing moisture and heat retention as well as soil aeration (Wang et al., 2019), promote crop root growth, and ultimately increase crop yield (Yuan et al., 2024). Meanwhile, the effects of BMFs on sugarcane rhizosphere microbial communities exhibit taxon-specific patterns, significantly increasing the diversity and abundance of bacteria and fungi in rhizosphere soils (Qi et al., 2020; Hu X. et al., 2025), while markedly reducing the abundance of pathogenic microorganisms (Wang C. et al., 2025). Different thicknesses of BMFs can regulate degradation rates and soil microclimatic conditions (Laycock et al., 2017), thereby generating distinct rhizosphere microhabitats throughout the crop growth period. These thickness-dependent differences influence both the timing and quantity of biodegradable carbon released into the soil while simultaneously altering soil moisture and temperature regimes (Zhang W. et al., 2025). Such changes alter environmental filtering and resource availability, thereby shaping microbial community assembly. Because bacteria and fungi differ markedly in their ecological strategies and substrate preferences, shifts in resource availability are expected to reshape cross-kingdom interaction networks and regulate the abundance of microbial functional genes involved in nutrient cycling, particularly those associated with carbon, nitrogen, and sulfur transformations. However, experimental evidence linking biodegradable mulch film thickness with microbial interaction networks and nutrient cycling functions under field conditions remains limited.

Although previous studies have demonstrated that BMFs can influence soil environments and crop performance, the ecological mechanisms linking mulch properties, rhizosphere microbial assembly, and plant productivity remain poorly understood. Based on ecological theories of environmental filtering, microbial community assembly, and ecosystem functioning, we hypothesized that (1) BMFs with different thicknesses would differentially affect sugarcane rhizosphere microbial communities due to their distinct degradation characteristics and effects on soil microhabitats; (2) BMF-associated environmental filtering would modify microbial community assembly processes and cross-kingdom interaction networks; and (3) these microbial changes would be associated with shifts in predicted nutrient cycling-related functional potentials, which may contribute to variations in sugarcane productivity. Therefore, this study aims to (1) evaluate the effects of BMFs on rhizosphere soil physicochemical properties and sugarcane agronomic traits; (2) elucidate the regulatory mechanisms by which BMFs of different thicknesses influence rhizosphere bacterial and fungal community composition, diversity, and community assembly processes; and (3) reveal the relationships among rhizosphere microbial community structure, predicted nutrient cycling potentials, soil physicochemical properties, and sugarcane yield.

## 2 Materials and methods

### 2.1 Site description and experimental design

The field experiment was conducted in a sugarcane-growing area of Gengma County, Lincang City, Yunnan Province, China (23°70′ N, 99°66′ E), which is characterized by a subtropical monsoon climate. The mean temperature of the hottest month is 23.2 °C, while that of the coldest month is 11.6 °C, and the mean annual precipitation is 1,303.6 mm. Precipitation during the rainy season (May–October) accounts for 86.6% of the annual total, with a mean relative humidity of approximately 70%, resulting in distinct wet and dry seasons; the experimental soil is a yellow-brown earth classified as a Chromic Luvisol according to the WRB system (IUSS Working Group WRB, 2022). Before experiment initiation, baseline soil samples were collected from the 0–40 cm soil layer. The baseline soil properties were pH 6.0, soil organic matter content of 18.4 g kg^−1^, and total nitrogen content of 1.2 g kg^−1^. The experiment compared three mulching treatments: (i) conventional polyethylene mulch film (PM, 0.008 mm thickness), (ii) PBAT/PLA-based biodegradable mulch film BTK (0.008 mm thickness), and (iii) PBAT/PLA-based biodegradable mulch film BTH (0.005 mm thickness). The treatments were arranged in a randomized complete block design with three replicates. A 1 m-wide buffer walkway was established between adjacent blocks. Each plot covered an area of 36 m^2^, with a row spacing of 1.2 m, a row length of 6 m, and five rows per plot. The planting density was uniform across treatments, with 120,000 buds ha^−1^. The sugarcane cultivar used in this study was ROC22. On 16 November 2019, a one-time basal fertilization was applied to meet nutrient requirements throughout the entire growth period, using a controlled-release fertilizer (23:13:18, N:P_2_O_5_:K_2_O) at a rate of 1,500 kg ha^−1^. To avoid fertilizer burn caused by direct contact between buds and fertilizer, the fertilizer was thoroughly mixed with fine soil in the planting furrow before placing the sugarcane setts. In August 2020, at harvest, sugarcane yield and related growth phenotypic traits were measured following the methods described by (Christina et al. 2024); (Dingre and Gorantiwar 2021).

### 2.2 Plant and soil sample collection and analysis

Agronomic traits were measured at sugarcane harvest in August 2020. Plant height of five representative sugarcane plants per plot was measured using a measuring rod, and stalk diameter was determined with a vernier caliper. After destructive sampling, the single stalk weight of five representative plants was measured and recorded. To measure the sucrose content, an Extech Portable Sucrose Brix Refractometer (Mid-State Instruments, San Luis Obispo, CA, USA) was used, and the calculation was performed using the following formula (Lin et al., 2013) (Equation 1):


$$\begin{array}{l} Sucrose\left(\%\right)=Brix\left(\%\right)\times 1.0825-7.703 \end{array}$$ (1)

The theoretical yield of sugarcane was estimated using the following Equations 2, 3:


$$\begin{array}{l} \begin{array}{cc} Singlestalkweight\left(kg\right) & ={\left(stalkdiameter\left(cm\right)\right)}^{2}\\ \times \left(stalkheight\left(cm\right)-30\right)\\ \times 1\left(g/cm^{3}\right)\times 0.7854/1000 \end{array} \end{array}$$ (2)

$$\begin{array}{lll} Theoreticalproduction\left(kg/hm^{2}\right) & = & singlestalkweight\left(kg\right)\\ \times & productivestemnumbers\left(hm^{2}\right) \end{array}$$ (3)

At the sugarcane harvest stage, rhizosphere soil samples (0–40 cm depth) were collected. For each treatment, nine biological replicates (*n* = 9) were collected. Each replicate consisted of rhizosphere soil collected from five randomly selected sugarcane plants within each plot using the five-point sampling method, resulting in a total of 27 soil samples. After excavation, loosely attached soil was removed by gently shaking the roots, and tightly adhering rhizosphere soil on the root surface was carefully collected using a sterile brush. The collected rhizosphere soils were placed in sterile bags and transported to the laboratory in ice boxes. Visible plant residues were removed, and the samples were homogenized before being divided into three portions for subsequent analyses. One portion was stored at 4 °C for the determination of soil moisture content and available nutrients. Another portion was air-dried and sieved for analysis of soil physicochemical properties. The final portion was stored at −80 °C for microbial DNA extraction and high-throughput sequencing (Duan et al., 2025). Soil pH was measured in a 1:2.5 (w/v) soil-water suspension using a pH meter (Mettler Toledo FE28, Switzerland). Soil organic matter (SOM) content was determined by the potassium dichromate oxidation method. Available nitrogen (AN) was measured using the alkaline hydrolysis diffusion method. Available phosphorus (AP) was extracted and determined via the molybdenum blue method. Available potassium (AK) was extracted and quantified by flame photometry (FP640, Shanghai, China) (Bao, 2000).

### 2.3 Soil microbial community characterization

Total genomic DNA was extracted from the experimental soil using the Power Soil DNA Isolation Kit (MoBio Laboratories Inc., Carlsbad, USA) according to the manufacturer's instructions. The bacterial 16S rRNA gene (V3-V4 region) was amplified with primers 338F 5′-ACTCCTACGGGAGGCAGCAG3′) and 806R 5′-GGACTACHVGGGTWTCTAAT3′), and the eukaryotic 18S rRNA gene was amplified with primers 528F 5′-GCGGTAATTCCAGCTCCAA3′) and 706R 5′-AATCCRAGAATTTCACCTCT3′). The 18S rRNA gene was selected as the marker because 18S rRNA sequencing provides broader coverage of eukaryotic microorganisms and minimizes potential biases associated with the use of fungal-specific primers. Following taxonomic annotation, non-fungal eukaryotic sequences were removed, and the remaining fungal sequences were retained for subsequent fungal community analyses. The amplification condition was 95 °C for 3 min, followed by 35 cycles of 95 °C for 30 s, 55 °C for 30 s, and 72 °C for 45 s, with a final extension at 72 °C for 10 min (GeneAmp 9,700, ABI, California CA, USA). PCR reaction was performed in triplicate in a 20-μL mixture containing 2 μL of 2.5 mM deoxyribonucleoside triphosphate (dNTPs), 4 μL of 5 × Fast Pfu buffer, 0.4 μL of Fast Pfu polymerase, 0.4 μL of each primer (5 μM), and template DNA (10 ng). PCR products were pooled, purified, and sequenced on an Illumina MiSeq PE300 platform (Illumina, USA).

Raw sequencing reads were processed using the QIIME 1 pipeline (v1.9.1). Low-quality reads and sequences shorter than 200 bp were filtered out. Chimeric sequences were identified and removed using UCHIME. Quality-filtered sequences were clustered into operational taxonomic units (OTUs) at a 97% similarity threshold using UCLUST. Taxonomic classification of bacterial OTUs was performed against the SILVA 132 database. Eukaryotic OTUs were classified using the PR2 (Protist Ribosomal Reference) database. OTUs classified as *Metazoa, Streptophyta*, or *Chlorophyta* were removed prior to fungal community analysis. These non-fungal sequences accounted for 25.31% of the total high-quality eukaryotic reads. Functional predictions of bacterial communities were performed using PICRUSt2 (v2.4.1) and FAPROTAX (v1.2.4) (Zhao W. et al., 2024). Fungal trophic modes and guilds were annotated using FUNGuild (v1.1) (Fang et al., 2022). The processed sequences were submitted to the Sequence Read Archive (SRA) of the National Center for Biotechnology Information (NCBI). Project identification number: PRJNA815949.

### 2.4 Microbial and statistical analysis

Alpha diversity indices (ACE, Chao1, Sobs) were calculated using the *vegan* (v2.6-2) and *microeco* (v0.14.0) packages in R software (v3.6.2). Beta diversity was assessed with Bray-Curtis distances and visualized by non-metric multidimensional scaling (NMDS). Permutational multivariate analysis of variance (PERMANOVA) with 999 permutations was used to test for significant differences in community structure among treatments (Park et al., 2025). Community assembly processes were assessed using neutral community model to estimate the relative importance of stochastic vs. deterministic processes (Liu et al., 2024). Microbial co-occurrence networks were constructed for each treatment using significant Spearman correlations (|ρ| > 0.6, *p* < 0.05, adjusted using the Benjamini-Hochberg false discovery rate correction) (Guo et al., 2025). Network topology properties were calculated using the *igraph* (v1.4.3) and *microeco* (v0.14.0) packages in *R* software (v3.6.2) (Zhao Z. et al., 2024). The roles of individual nodes (OTUs) within networks were classified into categories (peripherals, connectors, module hubs) based on within-module connectivity (Zi) and among-module connectivity (Pi). Similarity percentage analysis (SIMPER) was performed to identify the taxonomic groups contributing most to differences between treatments (Walker et al., 2022). Random Forest analysis was used to rank the importance of taxa in discriminating treatment groups (Gao et al., 2024). Linear discriminant analysis Effect Size (LEfSe) was employed to identify differentially abundant taxa with an LDA > 2. Relationships between microbial communities (structure, diversity, function, network properties), soil physicochemical variables, and sugarcane agronomic traits were examined using redundancy analysis (RDA), Procrustes analysis and Mantel tests implemented in the *vegan* package (Yan et al., 2025). All statistical analyses and visualizations were performed in *R* software (v3.6.2). Figures were generated using *ggplot2* (v3.4.1), *igraph*, and *Gephi* software (v0.10.1).

## 3 Results

### 3.1 Composition, diversity, and assembly characteristics of rhizosphere microbial communities

**Figure 1:**
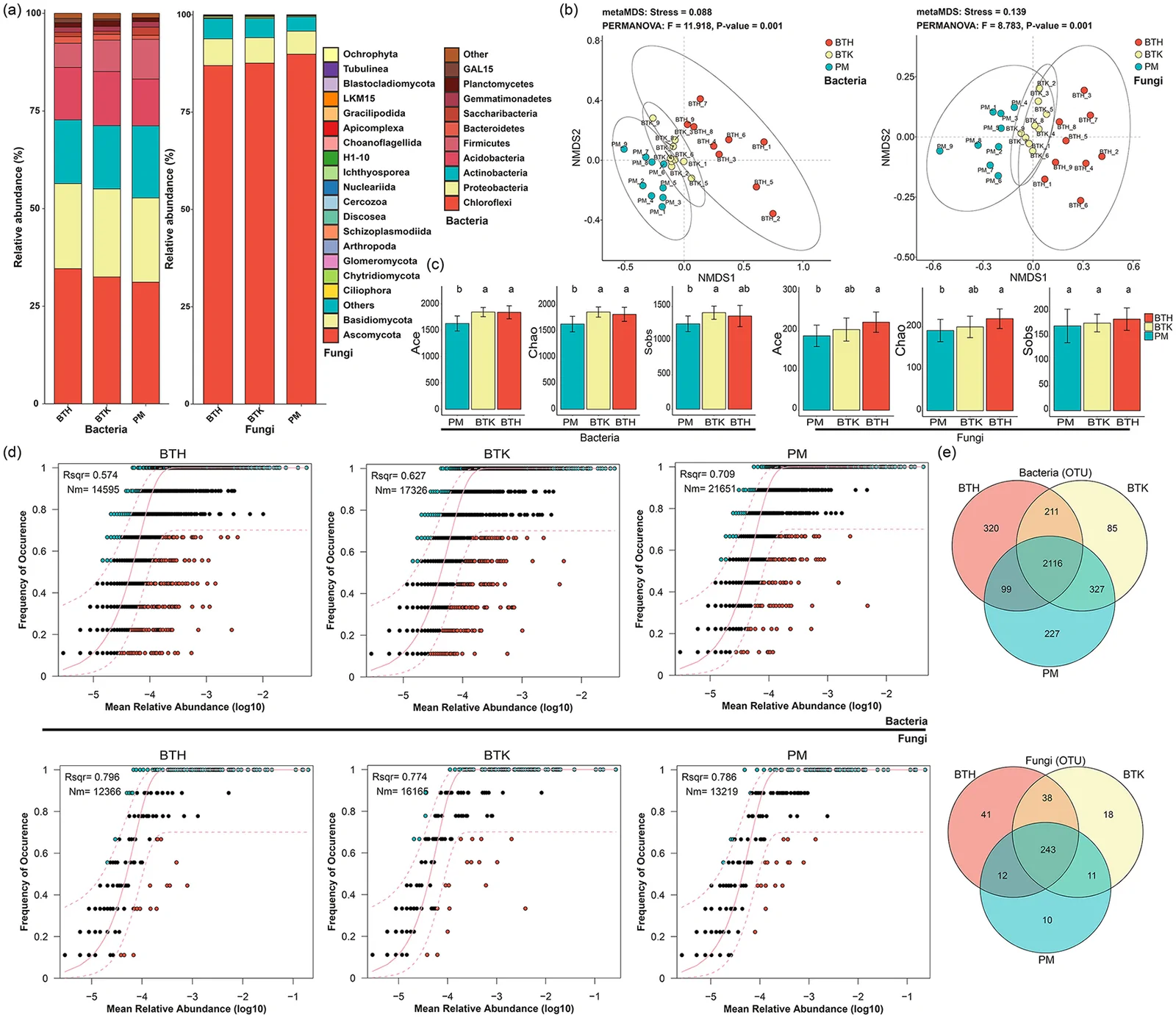
Mulch-driven changes in rhizosphere bacterial and fungal communities. **(a)** Taxonomic composition at the phylum level for bacteria and fungi. Bars show mean relative abundance (mean ± SE, *n* = 9). Phyla representing < 1% mean abundance are pooled as “Other.” **(b)** NMDS ordinations (Bray-Curtis distance) for bacterial and fungal communities. Points are sample centroids; ellipses show 95% confidence intervals around treatment centroids. **(c)** Alpha diversity indices (ACE, Chao and Sobs) for bacteria and fungi. Bars are mean ± SE (*n* = 9). Different letters denote significant differences among treatments at *p* < 0.05 by one-way ANOVA with LSD. **(d)** Neutral model fits for bacterial and fungal communities. Plots show observed occurrence frequency vs. mean relative abundance with Sloan neutral model fit (solid line) and 95% prediction intervals (dashed lines). **(e)** Venn diagrams of shared and unique OTUs among PM, BTK and BTH for bacteria and fungi. PM, conventional PE mulch; BTK and BTH, biodegradable mulches.

Conventional polyethylene (PE) mulch (PM, 0.008 mm) and biodegradable mulch films (BMFs, including BTK: 0.008 mm and BTH: 0.005 mm) significantly affected the composition, structural diversity, and community assembly processes of rhizosphere bacterial and fungal communities, exhibiting clear treatment-dependent differences (Figure 1). At the phylum level, rhizosphere microbial communities exhibited treatment-specific distributions under different mulching treatments (Figure 1a). *Chloroflexi, Proteobacteria, Actinobacteria, Acidobacteria*, and *Firmicutes* were the dominant bacterial phyla in the rhizosphere, collectively accounting for over 80% of the bacterial community. Compared with PM, BMFs (BTK and BTH) significantly increased the relative abundances of *Chloroflexi* and *Acidobacteria* (*p* < 0.05), while significantly decreasing the relative abundance of *Actinobacteria* (*p* < 0.05). *Ascomycota* and *Basidiomycota* were the overwhelmingly dominant fungal phyla, together accounting for more than 85% of the fungal community. BMFs treatments significantly increased the relative abundance of *Basidiomycota* (*p* < 0.05) and decreased that of *Ascomycota* (*p* < 0.05). Fungal phyla with relative abundances below 1%, such as *Chytridiomycota* and *Glomeromycota*, were grouped as others. Their total abundance under BMFs was slightly higher than PM, but the difference was not statistically significant.

Non-metric multidimensional scaling (NMDS) based on Bray–Curtis distances indicated that mulching type was the primary factor driving differentiation of rhizosphere microbial community structure (Figure 1b). The stress values for bacterial and fungal communities were 0.088 and 0.139, respectively, indicating reliable ordination results and clear clustering of samples, effectively reflecting differences among rhizosphere microbial communities. PM and BMFs treatments were completely separated, whereas BTK and BTH exhibited partial overlap but still formed distinct clusters. Permutational multivariate analysis of variance (PERMANOVA) further confirmed that mulching type significantly explained variations in bacterial (*F* = 11.918, *p* = 0.001) and fungal (*F* = 8.783, *p* = 0.001) community structures, consistent with the NMDS results. α-diversity analysis indicated that BMFs significantly enhanced rhizosphere microbial diversity, with differential effects observed between BTK and BTH (Figure 1c). For bacterial communities, ACE, Chao and Sobs indices were significantly higher under BTK and BTH compared with PM (*p* < 0.05), with BTK exhibiting greater bacterial diversity than BTH. Similarly, BMFs significantly increased fungal α-diversity (*p* < 0.05), with BTH showing higher ACE, Chao, and Sobs indices than BTK.

Neutral model analysis suggested that mulch type regulated microbial communities by modulating the balance between stochastic and deterministic assembly processes in the rhizosphere (Figure 1d). For bacterial communities, the model fit (Rsqr) for PM, BTK, and BTH treatments was 0.709, 0.627, and 0.574, respectively (all > 0.5), indicating a dominant role of stochastic processes. The Nm (metacommunity size × migration rate) decreased from PM to BTK to BTH (21651 → 17326 → 14595), indicating that as BMFs thickness decreased, dispersal limitation increased and deterministic processes (i.e., environmental filtering) became more prominent. For fungal communities, Rsqr values for all treatments exceeded 0.75 (PM: 0.786, BTK: 0.774, BTH: 0.796), indicating strong stochastic dominance. Nm values were highest under BTK (16165) and lowest under BTH (12366), suggesting that although fungal communities remained largely stochastic under BTH, dispersal limitation was significant, and communities were relatively isolated. Venn diagrams illustrated shared and unique operational taxonomic units (OTUs) among treatments (Figure 1e). In bacterial communities, 2116 OTUs were shared among all three treatments; the number of unique OTUs was BTH (320) > PM (227) > BTK (85). BTK shared a considerable proportion of OTUs with both PM and BTH, indicating its community composition was intermediate. In fungal communities, 243 OTUs were shared; BTH had the highest number of unique OTUs (41), whereas PM and BTK had fewer, further indicating that the thin biodegradable mulch film (BTH) exerted stronger taxon-specific selection, particularly on fungi.

### 3.2 Microbial community differences and biomarker taxa

**Figure 2:**
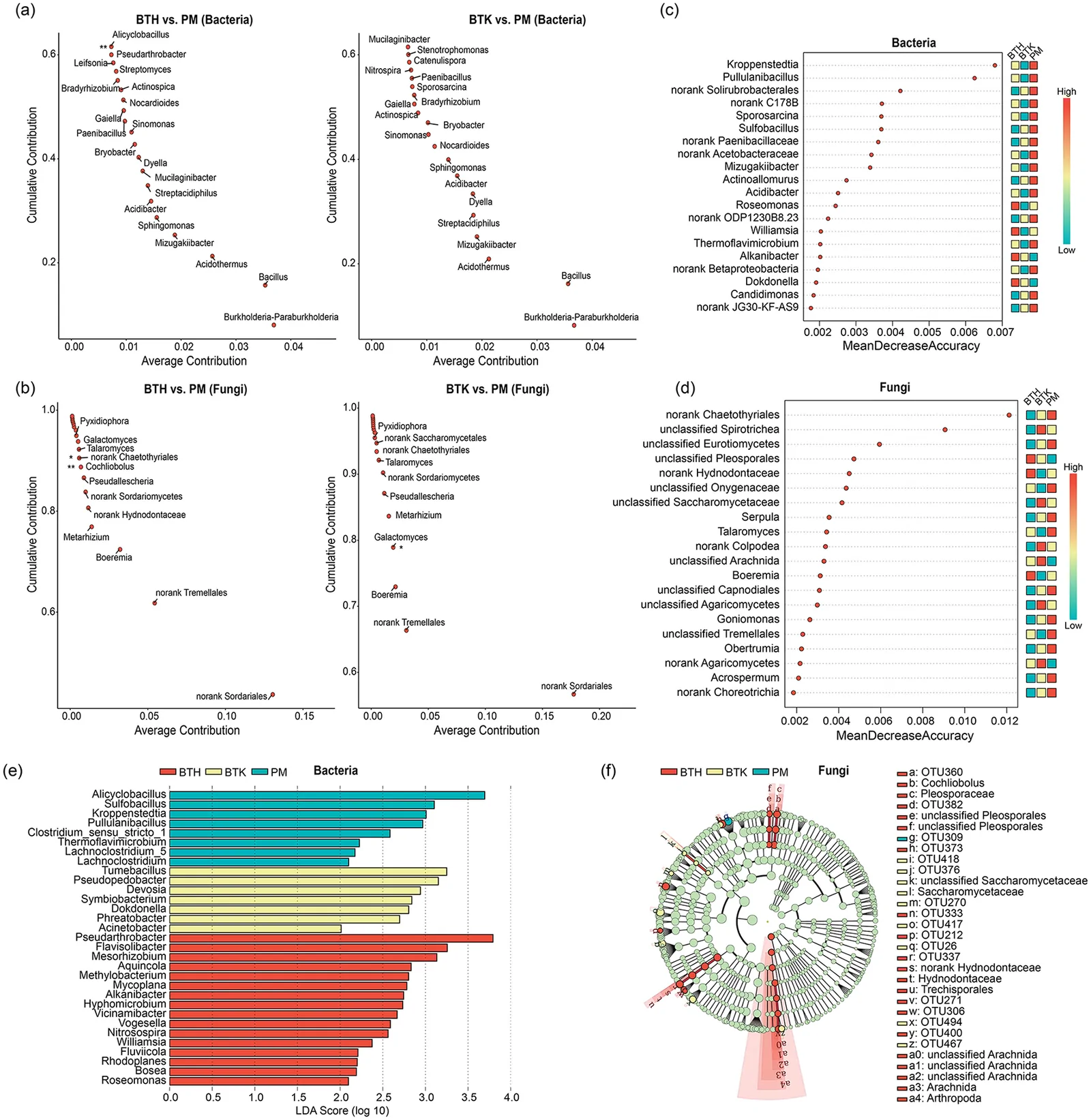
Taxa driving treatment differentiation and candidate biomarkers. **(a, b)** similarity percentage analysis (SIMPER) based on Bray-Curtis distance, showing cumulative contribution of top bacterial **(a)** and fungal **(b)** taxa to community differences between BTH vs. PM and BTK vs. PM (x-axis: average contribution of each taxon). **(c, d)** Random forest analysis for bacterial **(c)** and fungal **(d)** taxa, with x-axis representing MeanDecreaseAccuracy (indicator of taxon importance in distinguishing treatments) **(e)** LDA score bar plot for bacteria, showing differentially enriched taxa in PM, BTK, and BTH (x-axis: LDA score (log10), color-coded by treatment). **(f)** Fungal taxonomic maps shows fungal taxonomic units (labeled a-a4) across treatments.

Similarity percentage analysis (SIMPER) results identified the major taxa contributing to differences in microbial communities between PM and BMFs. In rhizosphere bacterial communities (Figure 2a), the differences between BTH and PM were primarily driven by changes in the abundances of *Alicyclobacillus, Pseudarthrobacter, Leifsonia*, and *Streptomyces*, whereas differences between BTK and PM were mainly attributed to *Mucilaginibacter, Stenotrophomonas, Catenulispora*, and *Nitrospira*. In rhizosphere fungal communities (Figure 2b), *Pyxidiophora, Galactomyces, Talaromyces*, and *Cochliobolus* (*p* < 0.01) were the main contributors to the differences between BTH and PM, whereas *Pyxidiophora, Talaromyces*, and *Galactomyces* (*p* < 0.05) played dominant roles in distinguishing BTK from PM. These results indicate that both BTH and BTK harbored bacterial and fungal community structures significantly different from PM, and the specific taxa driving these differences likely reflect the unique habitat conditions created by each BMF treatment.

Random forest analysis was further employed to identify microbial taxa with high discriminative power among treatments. Key rhizosphere bacterial genera distinguishing BTH from PM included *Sulfobacillus, Actinoallomurus, Alkanibacter*, and *Dokdonella* (Figure 2c), whereas the important taxa differentiating BTK from PM were *Kroppenstedtia, Pullulanibacillus, Sporosarcina, Mizugakiibacter, Acidibacter*, and *Thermoflavimicrobium*. *Roseomonas* and *Williamsia* were the key bacterial genera distinguishing BTH from BTK treatments. In rhizosphere fungal communities (Figure 2d), *Serpula, Goniomonas, Obertrumia*, and *Acrospermum* ranked highest in differentiating BTH from PM, *Talaromyces* played a key role in distinguishing BTK from PM, and *Boeremia* was the critical fungal genus distinguishing BTH from BTK.

Linear discriminant analysis effect size (LEfSe) analysis confirmed the treatment-specific enrichment patterns of bacterial taxa among the different mulching treatments (Figure 2e). PM was significantly enriched with *Alicyclobacillus* and *Sulfobacillus*; BTK was characterized by *Tumebacillus* and *Pseudopedobacter*; BTH specifically enriched *Pseudarthrobacter* and *Flavisolibacter*, with *Nitrosospira* also identified as a significantly enriched genus under BTH. Fungal taxonomic unit distribution and pairwise comparison results (Figure 2f) further revealed shared and treatment-specific fungal biomarkers. BTK was characterized by an unclassified genus within *Saccharomycetaceae*, whereas BTH enriched *Cochliobolus, Pleosporaceae, Hydnodontaceae, Trechisporales, Arachnida*, and *Arthropoda*. Overall, these results indicate that, compared with PM, BMFs selectively enrich specific bacterial and fungal taxa in the rhizosphere.

### 3.3 Associations of microbial communities with soil environmental factors and predicted functional potentials

**Figure 3:**
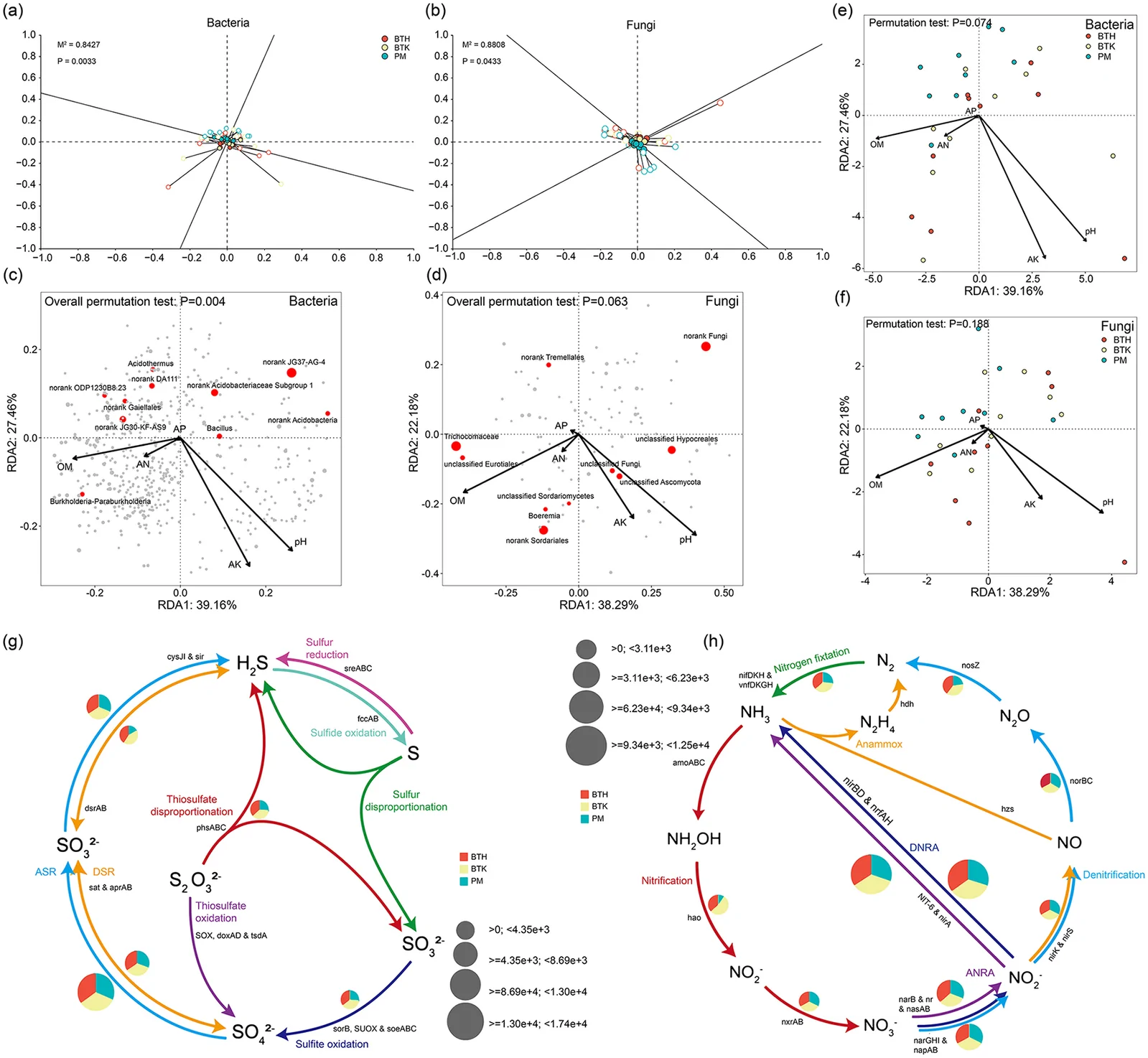
Associations between rhizosphere microbial communities, soil physicochemical factors, and sulfur/nitrogen cycling genes under different mulching treatments. **(a, b)** Procrustes analyses (based on PCoA ordinations) showing congruence between microbial community structure and soil physicochemical factors for bacteria **(a)** and fungi **(b)**. **(c, d)** RDA at genus level for bacteria **(c)** and fungi **(d)**; the top 10 genera (by loadings/contributions) are highlighted and labeled in red. Arrows indicate soil variables (AN, available nitrogen; OM, organic matter; AK, available potassium; AP, available phosphorus; pH, soil pH); arrow length and direction represent strength and sign of correlation with ordination axes. Axis labels report the percentage of constrained variance explained by each RDA axis. **(e, f)** RDA of treatment replicates constrained by measured environmental variables for bacteria **(e)** and fungi **(f)**; colors denote treatments (PM, BTK, BTH). Permutation test *P*-values for the constrained models are reported in each panel. **(g, h)** Analysis of soil sulfur **(g)** and nitrogen cycling **(h)**, based on KO abundance in the predicted bacterial functions from PICRUSt2. Circle size encodes the overall abundance range of the functional gene category; colored sectors indicate proportional contributions of each treatment.

Procrustes analysis indicated significant associations between soil physicochemical factors and microbial community structure, with response patterns varying among microbial groups (Figures 3a, b). The bacterial community structure showed significant concordance with soil factor ordination (M^2^ = 0.8427, *p* = 0.0033), suggesting that soil environmental conditions strongly influence bacterial community assembly. In contrast, fungal communities exhibited weaker concordance with soil factors (M^2^ = 0.8808, *p* = 0.0433), indicating that non-biotic drivers have a smaller influence on fungal community variation compared to bacteria.

Redundancy analysis (RDA) further revealed the constraining effects of key rhizosphere environmental factors on microbial community composition. At the bacterial genus level (Figure 3c), environmental factors had a highly significant effect on community variation (overall permutation test, *p* = 0.004), with RDA1 (39.16%) and RDA2 (27.46%) collectively explaining 66.62% of the constrained variation. Soil pH was the primary driver of bacterial communities, as indicated by the longest arrow; available potassium (AK) aligned closely with the pH vector, suggesting a synergistic effect on bacterial community composition. Soil organic matter (SOM) and ammonium nitrogen (AN) also exhibited strong coupled effects, whereas available phosphorus (AP) showed a relatively independent influence. *Bacillus* was significantly positively correlated with pH and AK, while *Acidothermus* was more enriched under conditions of higher SOM and AN. RDA analysis of fungal communities (Figure 3d) showed marginally significant effects of environmental factors (*p* = 0.063), with RDA1 (38.29%) and RDA2 (22.18%) collectively explaining 60.47% of the variation. pH, AK, and SOM were also the main factors influencing fungal composition. *Boeremia* was positively correlated with most environmental factors, whereas *Trichocomaceae* was negatively correlated with pH and AK but positively correlated with SOM. Constrained ordinations based on treatment replicates clearly illustrated the environmental drivers of community separation (Figures 3e, f). For bacterial communities, BTH and BTK samples were clearly separated from PM along the SOM and AN gradients, with the model showing marginal significance (*p* = 0.074). In fungal communities, PM and BTK samples partially overlapped, whereas BTH samples were more dispersed. These results indicate that pH, SOM, and AK are key environmental factors distinguishing PM from BMFs treatments.

Functional prediction using PICRUSt2 suggested treatment-dependent differences in the potential abundances of sulfur and nitrogen cycling genes under different mulching treatments. For sulfur cycling (Figure 3g), PM exhibited lower predicted abundances of gene families associated with ${\text{H}}_{2}\text{S}-{\text{SO}}_{3}^{2-}$ transformation (dsrAB), thiosulfate disproportionation (phsABC), and sulfite oxidation (sorB, SUOX, and soeABC), whereas BMF treatments (BTK and BTH) showed higher predicted abundances of these gene families. For nitrogen cycling (Figure 3h), PM exhibited lower predicted abundances of gene families involved in denitrification (nosZ) and nitrification (hao), whereas BTK showed higher predicted abundances of these gene families as well as nitrogen fixation gene families (nifDKH and vnfDKGH). Gene families associated with dissimilatory nitrate reduction to ammonium (DNRA; nirBD and nrfAH) were also predicted to be more abundant under BMF treatments. Compared with PM, BMF treatments also exhibited higher predicted abundances of gene families involved in the Wood–Ljungdahl pathway and acetoclastic methanogenesis, with similar trends observed for methylotrophic methanogenesis and methanol oxidation pathways (Supplementary Figure S6a). These results represent predicted community functional potential inferred from marker-gene profiles and warrant further validation using approaches such as qPCR or metagenomic analyses.

### 3.4 Characteristics of rhizosphere bacteria–fungi co-occurrence networks under different mulching treatments

**Figure 4:**
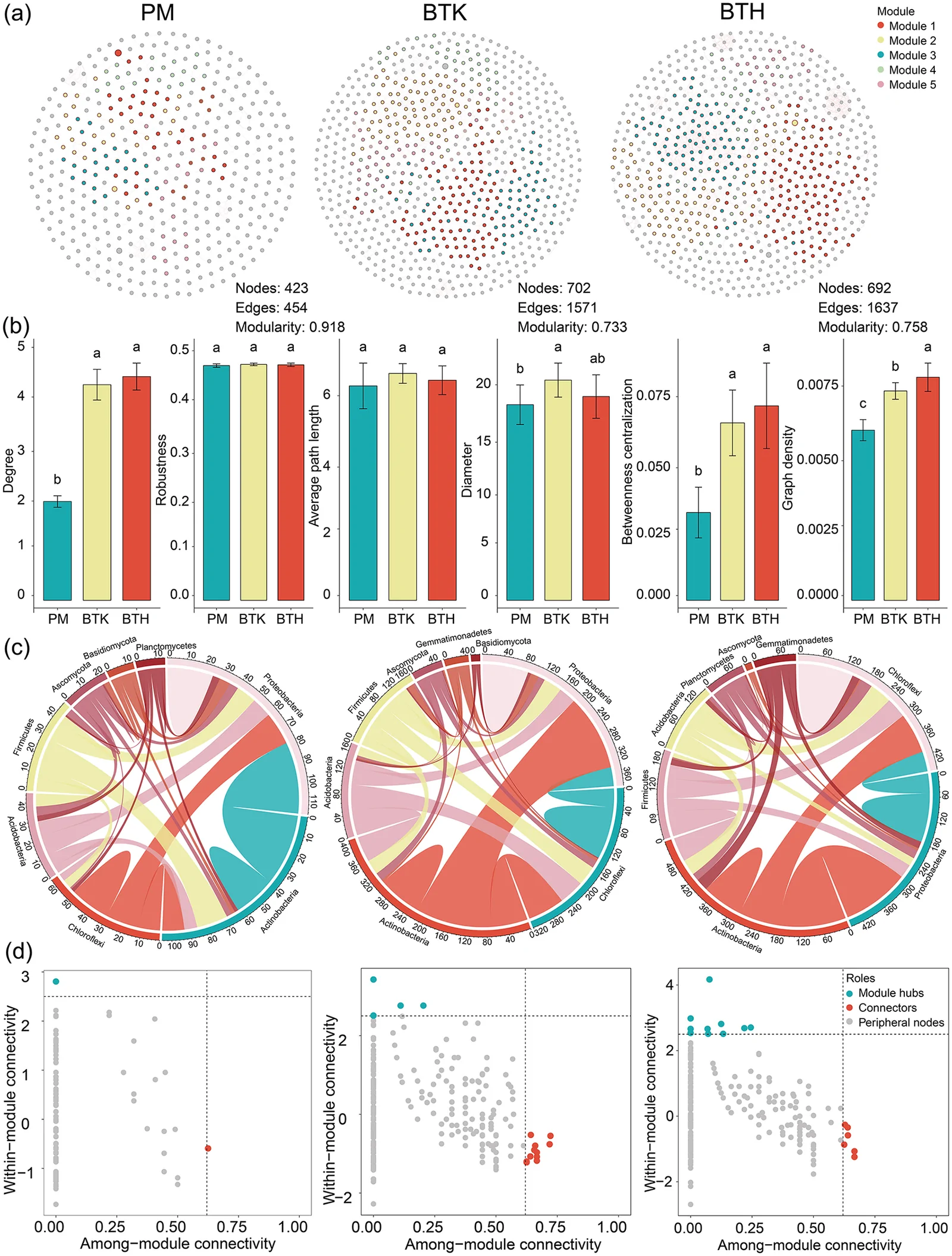
Rhizosphere bacterial-fungal co-occurrence network characteristics under different mulching treatments. **(a)** Bacteria-fungi co-occurrence networks for PM, BTK, and BTH; nodes represent operational taxonomic units (OTUs), edges represent significant correlations (Spearman's > 0.6, *p* < 0.05), and colors denote the top 5 modules; under show node number, edge number, and modularity index for each network. **(b)** Six network metrics summarizing topology differences among treatments: Degree (average node connections), Robustness (network stability), Diameter (longest shortest path), Average path length (network complexity), Graph density (edge proportion), and Betweenness centralization (central node reliance); error bars represent standard errors (*n* = 9), and different lowercase letters indicate significant differences among treatments at *p* < 0.05. **(c)** Linkage degrees of the top 8 dominant bacterial and fungal phyla across treatments (phylum names on axis). **(d)** Node role classification based on Zi (within-module connectivity) and Pi (among-module connectivity); dots represent nodes, colors distinguish node types.

To explore the effects of different mulching treatments on rhizosphere microbial interactions, bacteria–fungi co-occurrence networks were constructed based on Spearman correlation analysis (|ρ| > 0.6, *p* < 0.05) (Figure 4). The network topologies differed markedly among PM, BTK, and BTH treatments (Figure 4a). The PM network contained 423 nodes and 454 edges, with a modularity index of 0.918, exhibiting an isolated interaction pattern. The BTK network had 702 nodes and 1,571 edges, with a modularity index of 0.733. The BTH network comprised 692 nodes and 1,637 edges, with a modularity index of 0.758. Both BTK and BTH networks displayed more complex connectivity than PM. Notably, compared with PM (83.70%), the BTH network showed a significant increase in positive links (89.62%), whereas the BTK network exhibited the highest proportion of negative correlations (10.37% higher than PM), indicating differential regulation of microbial interactions by distinct BMFs (Supplementary Table S3).

Network topology metrics further quantified treatment-specific ecological strategies (Figure 4b). The average degree and betweenness centralization were highest in the BTH network, and both BMFs treatments were significantly higher than PM (*p* < 0.05), reflecting enhanced species connectivity and dependence on key nodes under BMFs. Network robustness and average path length did not differ significantly among treatments, although BTK had a relatively higher average path length. A similar trend was observed for network diameter. Graph density was highest under BTH and lowest under PM (*p* < 0.05), indicating that BMFs strengthened species associations within the microbial network, with BTH exhibiting the densest interactions. Moreover, the clustering coefficient in BTH was higher than in PM and BTK, indicating stronger local node aggregation; BTK exhibited the highest heterogeneity, highlighting greater diversity in node connectivity patterns; centralization in BTH was significantly higher than PM and BTK (Supplementary Table S3), further confirming stronger network reliance on core nodes under BTH, consistent with the betweenness centralization results.

Phylogenetic association analysis at the phylum level revealed differences in interaction patterns among treatments (Figure 4c). In PM, the fungal *Ascomycota* exhibited strong associations with bacterial *Proteobacteria* and *Acidobacteria*, whereas connections between *Actinobacteria* and *Chloroflexi* were relatively scattered. BMFs treatments significantly enhanced cross-domain interactions between fungal *Basidiomycota* and bacterial *Proteobacteria* and *Actinobacteria*, while strengthening intra-domain connections among bacterial groups such as *Chloroflexi* and *Acidobacteria*, indicating that BMFs promote both cross-domain and intra-domain microbial interactions. Node role classification based on within-module connectivity (Zi) and among-module connectivity (Pi) showed (Figure 4d) that PM was dominated by peripheral nodes, with key hub taxa including *Acidothermus* and *Alicyclobacillus*. BTK was dominated by connector nodes, with module hubs including *Tumebacillus, Pedobacter*, and *Rhizomicrobium*. BTH was dominated by module hubs, including *Acidothermus, Bacillus, Pseudonocardia*, and *Gemmatimonas*. These results indicate that BMFs, particularly BTH, significantly increased the number and diversity of key regulatory taxa in the network.

### 3.5 Associations among soil factors, sugarcane agronomic traits, and microbial indicators

**Figure 5:**
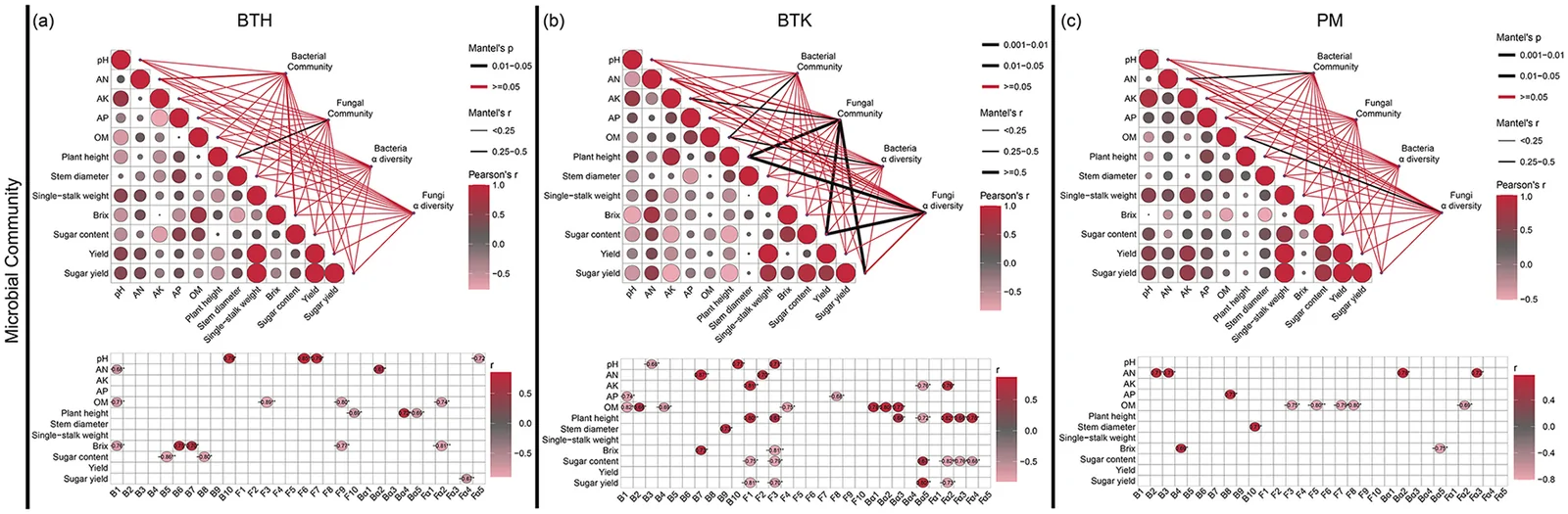
Mantel test analysis of relationships among soil physicochemical properties, sugarcane core agronomic traits, and rhizosphere microbial indicators under different mulching treatments. **(a–c)** Relationships between the above variables and microbial communities, including bacterial community, fungal community, bacterial α-diversity, and fungal α-diversity, where **(a)** corresponds to the BTH treatment, **(b)** to the BTK treatment, and **(c)** to the PM treatment. Circles represent Mantel test data points; Mantel *p*-value ranges are distinguished by line colors; Mantel *r*-value ranges are distinguished by line thickness. Pearson's r values are accompanied by significance labels: *p* < 0.05 (*) and *p* < 0.01 (**). AN, available nitrogen; AK, available potassium; SOM, soil organic matter; AP, available phosphorus; Brix, sugarcane sugar degree; Single-stalk Weight, sugarcane single-stalk dry weight; Stem Diameter, sugarcane stem diameter; Yield, sugarcane fresh yield; Sugar Yield, sugarcane sugar production; Sugar Content, sugarcane sugar percentage.

Mantel tests revealed complex associations among soil physicochemical properties, key sugarcane agronomic traits, and rhizosphere microbial indicators (functional, community, and network features) under different mulching treatments (Figure 5). Regarding microbial functions (Supplementary Figures S7a–c), the correlation between functional prediction matrices based on PICRUSt2, FAPROTAX, and FUNGuild and soil factors or agronomic traits exhibited notable treatment-specific differences. Under BTH (Supplementary Figure S7a), FAPROTAX functions were significantly positively correlated with Brix (*p* < 0.05), while FUNGuild functions were significantly positively correlated with plant height (*p* < 0.01). In BTK (Supplementary Figure S7b), the PICRUSt2 functional matrix was significantly negatively correlated with SOM (*p* < 0.01), whereas FAPROTAX functions were significantly positively correlated with SOM content (*p* < 0.05). Under PM (Supplementary Figure S7c), PICRUSt2 functions were significantly positively correlated with sugarcane Brix (*p* < 0.05), FAPROTAX functions were significantly positively correlated with AN (*p* < 0.05), and FUNGuild functions were significantly negatively correlated with SOM (*p* < 0.05).

Responses of microbial community structure and diversity indices to different factors also varied by treatment. Bacterial community structure in BTK was significantly negatively correlated with SOM (Figure 5b). Fungal community structure was significantly positively correlated with AK and plant height, but significantly negatively correlated with sugar content and sugar yield. Bacterial α-diversity was significantly positively correlated with SOM (*p* < 0.05), while fungal α-diversity exhibited a similar pattern to fungal community structure. In BTH, fungal community structure was significantly negatively associated with plant height (Figure 5a). Under PM, bacterial community structure was significantly positively correlated with AN (Figure 5c), whereas fungal community structure and α-diversity were significantly negatively correlated with SOM (*p* < 0.05).

Associations between microbial co-occurrence network topological properties and soil or sugarcane agronomic factors further revealed treatment-specific differences. In PM (Supplementary Figure S7f), betweenness centralization was significantly negatively correlated with single stalk weight, cane yield, and sugar yield (*p* < 0.01). In BTH (Supplementary Figure S7d), betweenness centralization was also significantly negatively correlated with plant height (*p* < 0.05). Notably, in BTK (Supplementary Figure S7e), graph density was significantly positively correlated with stalk diameter (*p* < 0.05).

### 3.6 Integrated effects of biodegradable mulches on rhizosphere processes and sugarcane productivity

**Figure 6:**
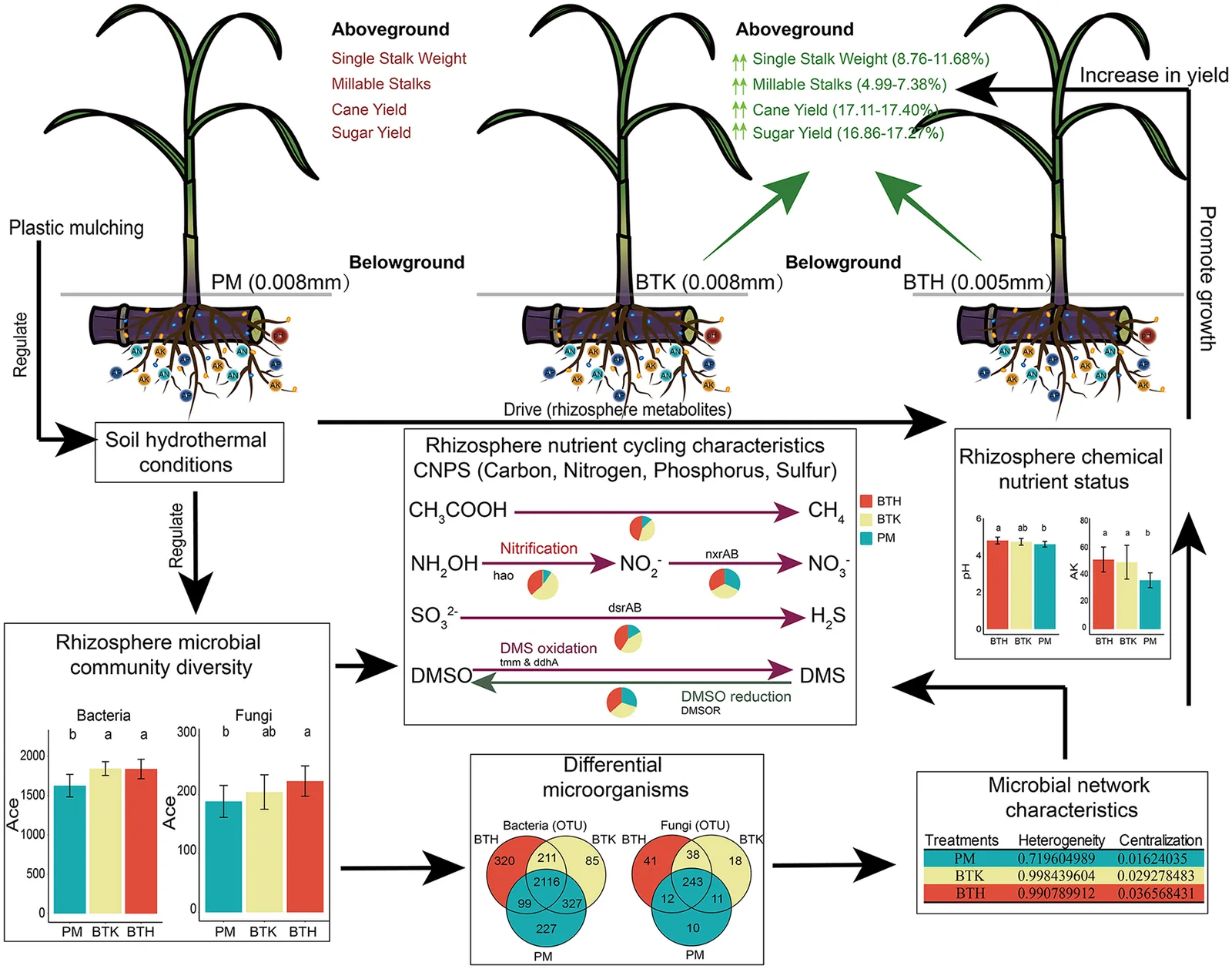
Conceptual diagram of rhizosphere microbial-driven yield-promoting mechanisms under biodegradable mulches. Mulch treatments include conventional PE mulch (PM, 0.008 mm) and biodegradable mulches (BTK, 0.008 mm; BTH, 0.005 mm), distinguished by thickness labels. Regulation of soil hydrothermal conditions under BMFs significantly altered rhizosphere pH and available potassium (AK), which were linked to shifts in microbial α-diversity. These microbial responses, together with enrichment of unique operational taxonomic units (OTUs; illustrated by Venn diagrams) and changes in network properties (microbial co-occurrence patterns), collectively enhanced rhizosphere carbon, nitrogen, phosphorus, and sulfur cycling. The integrated belowground processes ultimately translated into improved aboveground sugarcane performance.

Integrated analysis revealed a cascading pathway linking biodegradable mulch films (BMFs), rhizosphere environmental modification, microbial community differentiation, nutrient cycling potential, and sugarcane productivity (Figure 6). Compared with conventional polyethylene mulch (PM), BMFs improved rhizosphere soil conditions, particularly by increasing soil pH and available potassium (Supplementary Table S2), which contributed to the differentiation of bacterial and fungal communities. These microbial shifts were further associated with enhanced predicted functional potentials involved in carbon, nitrogen, and sulfur cycling, as well as more complex bacteria–fungi interaction networks, indicating improved ecological functioning of the rhizosphere. The regulation of belowground microbial processes was ultimately reflected in sugarcane growth performance. Compared with PM, BMFs increased single stalk weight by 8.76%−11.68%, millable stalk number by 4.99%−7.38%, cane yield by 17.11%−17.40%, and sugar yield by 16.86%−17.27% (Supplementary Table S1). Among the two biodegradable mulches, the thinner BTH treatment induced greater differentiation in microbial community composition and stronger shifts in predicted functional potentials, whereas the thicker BTK treatment maintained relatively stable microbial community responses. Collectively, these results suggest that BMFs promote sugarcane productivity through a thickness-dependent cascade of soil environment improvement, microbial ecological regulation, and nutrient cycling enhancement.

## 4 Discussion

### 4.1 Biodegradable mulch films selectively regulate sugarcane rhizosphere microbial community composition

Compared with conventional PE mulch (PM), biodegradable mulch films (BTK and BTH) significantly altered the phylum- and genus-level composition of sugarcane rhizosphere microbial communities, and Permutational Multivariate Analysis of Variance (PERMANOVA) confirmed that mulch type was a key driver of community differentiation (Figure 1b). This finding is consistent with previous studies (Yang et al., 2023), showing that biodegradable mulches can significantly drive rhizosphere microbial community structure differentiation by modifying the soil microenvironment (Zhang et al., 2024b). Moreover, it has been reported that PBAT-based biodegradable mulch can promote sugarcane nutrient metabolism by modifying rhizosphere microbial communities (Wang F. et al., 2024), likely because biodegradable mulches are primarily degraded by microbial activity in the soil, where microbes decompose the polymer structures and release organic hydrolysates (Hao et al., 2024). These products serve as exogenous carbon sources for soil microbes, altering their metabolic preferences and stimulating the proliferation of functional microbial groups involved in carbon cycling (Liu et al., 2026).

At the phylum level, BMFs increased the relative abundance of *Chloroflexi* by 18.2%−25.6% and *Acidobacteria* by 12.5%−19.8% (*p* < 0.05) (Figure 1a). This result is consistent with a previous study in cabbage fields, where biodegradable mulches increased *Acidobacteria* abundance by 19.82% (Liang et al., 2025). Other studies have indicated that *Chloroflexi* is associated with soil carbon accumulation under biodegradable mulch microplastics (Chen et al., 2024). Mechanistically, the enrichment of *Chloroflexi* and *Acidobacteria* may be related to the altered rhizosphere environment following BMFs degradation. Changes in soil physicochemical properties, including pH, organic matter content, and nutrient availability, can strengthen environmental filtering and favor oligotrophic taxa adapted to relatively stable resource conditions. Consistent with this interpretation, rhizosphere pH increased from 6.1 under PM to 6.4 under BTH treatment (Supplementary Table S2), potentially providing more suitable conditions for the persistence of *Chloroflexi* and *Acidobacteria*.

BMFs also significantly reduced the relative abundance of *Actinobacteria* by 15.7%−21.4% and *Ascomycota* by 18.9%−25.3% (*p* < 0.05) (Figure 1a). This differs from some previous reports showing that PE microplastics (PE-MPs) had no significant effect on *Actinobacteria* abundance in soil (Hu X. et al., 2023). Other studies have suggested that biodegradable mulches can reduce plastic residues, alter soil phosphorus fractions, and regulate key phosphorus cycling genes and enzyme activities, thereby potentially favoring oligotrophic bacteria such as *Actinobacteria* (Liu et al., 2026). However, the decline of *Actinobacteria* observed in our study indicates that BMF-induced microbial shifts were not solely determined by general oligotrophic characteristics but also depended on species-specific ecological niches and competitive interactions. The increased dominance of *Acidobacteria* and *Chloroflexi* under BMF treatments may have altered resource utilization patterns and niche availability, thereby reducing the relative advantage of *Actinobacteria* in the sugarcane rhizosphere. The contrasting responses among oligotrophic groups suggest that BMF-induced niche differentiation may favor some taxa while suppressing others depending on their ecological strategies and competitive interactions.

### 4.2 Mulch thickness modulates microbial diversity and community assembly processes

Non-metric multidimensional scaling (NMDS) showed that PM and BMFs samples were completely separated, with BTK and BTH forming independent clusters (Figure 1b). BMFs significantly enhanced sugarcane rhizosphere microbial α-diversity (Wang C. et al., 2025; Liu et al., 2026). For bacterial α-diversity (ACE, Chao, and Sobs indices), the order was BTK > BTH > PM, whereas fungal α-diversity index was BTH > BTK > PM, indicating that thicker BMFs better enhanced bacterial diversity, while thinner BMFs favored fungal diversity maintenance. All indices differed significantly between BMFs and PM (*p* < 0.05) (Figure 1c). This is consistent with previous conclusions that biodegradable microplastics (Bio-MPs) significantly increase rhizosphere bacterial α-diversity (Chao1, ACE, Shannon, Simpson) (Meng et al., 2023), contrasting with studies showing that microbial α-diversity on MPs surfaces is generally lower than in surrounding soil (Zhang X. et al., 2025). These differences may be associated with additional carbon inputs from BMF degradation products and sugarcane root exudates, which together could influence microbial resource availability (Xu et al., 2024). Additionally, Bio-MPs produced during BMFs decomposition have rough surfaces that enhance microbial colonization (Bai et al., 2025), while DOC released during BMF degradation has been reported to potentially increase microbial biomass carbon (Chen et al., 2024; Yu Y. et al., 2025), providing more resources for diverse species and expanding the soil labile carbon pool.

Neutral model analysis indicated that bacterial and fungal community assembly under different mulches was predominantly governed by stochastic processes. However, BMFs significantly reduced the Nm, increasing the relative contribution of deterministic processes (Figure 1d). This is consistent with previous reports (Liu et al., 2026) showing that both PE and biodegradable mulches are dominated by stochastic assembly. Previous studies have suggested that BMF application may influence microbial habitat conditions during degradation, potentially reducing ecological drift and increasing the relative contribution of deterministic processes (Yu H. et al., 2025). In this study, Nm was lowest under BTH, suggesting that thinner mulch may have induced stronger environmental selection pressures compared with thicker mulch. This pattern could be associated with thickness-dependent differences in degradation dynamics and resource availability; however, these mechanisms require further validation through direct measurements of mulch degradation and soil environmental changes. Compared with thicker BTK mulch, the distinct physicochemical characteristics of BTH may generate different resource availability patterns and microbial selection pressures, potentially contributing to the formation of treatment-specific microbial niches, where microbial colonization and resource availability differ from the surrounding soil matrix. Meanwhile, differences in the release patterns of biodegradable intermediates between mulch thickness treatments may influence the availability of organic substrates and consequently affect microbial recruitment patterns. Such habitat differentiation may increase environmental filtering intensity and contribute to the observed shift toward deterministic assembly, thereby reducing the influence of dispersal processes and resulting in lower Nm values under BTH treatment. This interpretation is consistent with previous findings that increased microhabitat heterogeneity enhances environmental filtering and promotes deterministic microbial community assembly (Ji et al., 2024). The contrasting assembly patterns between bacterial and fungal communities may be related to their different ecological strategies and dispersal mechanisms. Bacteria are generally more sensitive to local changes in soil physicochemical conditions due to their reliance on cell-level dispersal and immediate resource availability, potentially resulting in stronger environmental filtering under thinner BMF treatment. In contrast, fungi possess extensive hyphal networks that facilitate spatial exploration and resource acquisition across heterogeneous soil microsites, while their extracellular enzyme systems enable flexible utilization of complex substrates. These characteristics may enhance fungal community resilience to mulch-induced environmental fluctuations, allowing stochastic processes to remain dominant across different treatments. Venn diagram analysis showed that BTH had the highest number of unique OTUs, with BTK and PM having fewer (Figure 1e). Previous studies suggest that thinner BMFs promote soil microbial richness and diversity more than thicker ones (Wang S. et al., 2024; Liang et al., 2025). Although the abundance of microplastic-associated unique microbes is lower than in soil, mulch type significantly regulates the composition of unique taxa. This may be because the faster degradation rate of thin BTH mulch creates transient and heterogeneous rhizosphere niches, selectively enriching microorganisms adapted to fluctuating resource conditions, whereas thicker BTK mulch provides a relatively stable habitat with slower resource turnover, the specific contribution of degradation dynamics requires further investigation (Xia et al., 2025). Our results demonstrate that different BMF thicknesses altered dominant microbial taxa and community differentiation patterns, with BTH showing a stronger association with unique microbial assemblage formation.

### 4.3 BMF-driven microbial community shifts are associated with environmental filtering and predicted nutrient cycling-related functional potentials

Joint analysis using SIMPER, Random Forest, and LEfSe revealed the core differential microbial taxa between BMFs and PM treatments, showing significant treatment specificity (Figure 2). The selective enrichment characteristics of different BMFs treatments (BTH, BTK) were pronounced, and their ecological functional traits further elucidated the intrinsic mechanisms by which BMFs regulate the rhizosphere microenvironment. In the bacterial community, the differences between BTH and PM were primarily driven by *Alicyclobacillus, Flavisolibacter*, and *Pseudarthrobacter*, consistent with previous findings (Luo et al., 2022; Zhang Y. et al., 2022; Wang et al., 2023). These taxa are closely associated with extracellular enzyme secretion, carbohydrate metabolism, and organic pollutant degradation (Chai et al., 2025), and their enrichment confirms that BTH mulch selectively filters function-specific microbial taxa by altering microhabitats (Ni et al., 2021). The core differential taxa between BTK and PM included *Nitrospira* and *Stenotrophomonas*; the former functions as a key nitrifier in the nitrogen cycle, while the latter enhances organic matter decomposition, reflecting the targeted regulation of nutrient cycling by BTK (Luo et al., 2020). Moreover, the enrichment of the key fungal differential genus *Talaromyces* under BTK may be related to the stable microhabitats formed by the slower degradation of thicker BMFs, with some strains confirmed to secrete plant growth-promoting substances (Yang et al., 2023). Random Forest further identified highly discriminative taxa such as *Sulfobacillus* and *Actinoallomurus*, with the sulfur-oxidizing activity of *Sulfobacillus* enhancing soil nutrient availability (Zhang D. et al., 2025). The taxa differentiating BTH and BTK (*Roseomonas* and *Boeremia*) further indicate that BMFs of different thicknesses can selectively enrich functionally distinct microbial groups by shaping specific microhabitats. In our study, LEfSe results indicated the specific enrichment of *Nitrosospira* in BTH, further confirming the promotive effect of BMFs on nitrification (Huang S. et al., 2023). Conversely, PM treatment significantly enriched *Alicyclobacillus*, which is better adapted to the SOM dominated environment under PE mulch (Fan P. et al., 2024). These differences in bacterial distribution essentially result from the contrasting degradation properties and physicochemical microenvironments between BMFs and PM (Shangguan et al., 2024).

The fungal community differences were even more pronounced, with saprotrophic fungi such as *Pyxidiophora* and *Galactomyces* being core contributors in comparisons among BMFs and PM (Figure 2b). These fungi secrete cellulases and laccases to degrade lignocellulose, accelerating carbon cycling and nutrient release (Sánchez, 2009). The enrichment of *Cochliobolus* and *Pleosporaceae* under BTH treatment has been previously reported; LEfSe analysis of sugarcane rhizosphere diversity identified *Cochliobolus* as a highly important fungal taxon under bio-fertilizer treatment, indicating that thinner BMF coverage is more conducive to improving the microbial habitat for sugarcane (Liu et al., 2022). Notably, *Talaromyces* was a key taxon in both BMFs treatments; its phosphate-solubilizing ability and plant growth-promoting functions provide a microbial explanation for the agronomic advantages of BMFs (Chandra et al., 2025). The degradation of BMFs alters soil pH, SOM content, and enzyme activity, while the released MPs reshape community structure through particle size and concentration effects (Hu et al., 2024). In contrast, PM treatment enriches stress-tolerant taxa capable of degrading recalcitrant carbon through the formation of stable biofilms (Gao and Sun, 2021). This study indicates that the unique microhabitats created by BMFs are key drivers of microbial community changes.

RDA results indicated that soil pH, AK, and SOM are the primary drivers shaping microbial community structures under PM and BMFs coverage, with bacteria being more sensitive to these factors than fungi (Figure 3). Previous studies have shown that long-term BMFs coverage significantly increases soil nutrient content such as AK and SOM, and improves soil hydrothermal microenvironments, thereby significantly enhancing soil enzyme activity and indirectly influencing crop growth (Shangguan et al., 2024). pH is a major driver of bacterial community composition, diversity, and function, with bacterial richness generally increasing from acidic to neutral pH (Zhou et al., 2024). In this study, BTH treatment significantly increased soil pH (Supplementary Table S2) and concurrently enhanced microbial community diversity (Figure 1c), mitigating the acidification risk caused by PM. *Bacillus* was positively correlated with pH; previous research indicates that *Bacillus* possesses adaptive tolerance to pH stress, and elevated pH in acidic environments promotes its growth and metabolism (Ren et al., 2025). SOM can enhance microbial abundance and diversity, support the growth of copiotrophic bacteria, and improve soil aggregation, which may be related to differences in carbon source availability under different mulch treatments (Chen et al., 2024; Song et al., 2024; Xu et al., 2024). Our results show that *Acidothermus* and *Trichoderma* are more likely to be enriched in environments with higher SOM, possibly because *Acidothermus* has long been considered a genus involved in organic matter decomposition across various soil environments, particularly under acidic stress conditions (Mendonca et al., 2025). The abundance of *Trichoderma* is positively correlated with SOM, indicating that higher SOM levels support greater *Trichoderma* presence (Chen et al., 2025). Consistent with previous studies, the colonization environment is the primary factor affecting microbial composition, followed by mulch material and degradation duration (Deng et al., 2025; Hu et al., 2022).

The predicted functional profiles of differential microbial taxa under different mulch treatments, together with changes in soil nutrient availability, were associated with shifts in microbial functional potentials related to nutrient cycling. PICRUSt2 functional prediction indicated that BMFs were associated with increased nutrient cycling-related functional potentials, and methanogenesis-related pathways (Figures 3g–h), forming a synergistic regulatory effect between taxa and genes, consistent with previous findings (Shi et al., 2025). In terms of sulfur cycling, BMFs significantly increased the abundance of functional genes such as dsrAB and phsABC, which are involved in key processes like hydrogen sulfide oxidation and thiosulfate disproportionation (Vigneron et al., 2021). The easily degradable organic carbon released during BMFs degradation provides sufficient carbon for sulfur-cycling microbes, while the slight increase in rhizosphere pH (Supplementary Table S2) alleviates inhibition of sulfur-cycling enzyme activity in acidic conditions (Anza et al., 2021), collectively promoting sulfur activation and cycling, supplying more available sulfur nutrients for sugarcane growth. During nitrogen cycling, BTK treatment significantly enriched genes related to nitrogen fixation (nifDKH), nitrification (hao), and denitrification (nosZ), which was directly associated with increased abundance of nitrogen-fixing taxa (e.g., *Rhizomicrobium*) and nitrifying taxa (e.g., *Nitrosospira*) under this treatment (Figures 2e–d). The thicker BTK mulch degrades slowly, continuously releasing carbon while maintaining a stable rhizosphere hydrothermal environment, providing favorable conditions for the colonization and metabolism of nitrogen-cycling microbes. In contrast, BTH treatment enriched genes related to denitrification (nosZ) and dissimilatory nitrate reduction to ammonium (DNRA), likely due to the pulse-like carbon input caused by the rapid degradation of the thinner mulch (Xu et al., 2024). Moreover, BMFs enriched genes related to the WL pathway and methanogenesis, suggesting potential regulation of methane production and acetate synthesis processes. Other studies have reported that the use of BMFs can significantly increase greenhouse gas emissions (Hao et al., 2024; Liang et al., 2025), indicating that BMFs may enhance soil carbon sequestration by participating in soil carbon cycling (Zhou et al., 2023). In contrast, PM treatment, lacking degradable carbon inputs, exhibited lower abundance of sulfur- and nitrogen-cycling functional genes, limiting soil nutrient transformation efficiency, which functionally explains the superior soil fertility and crop growth potential under BMFs treatment.

### 4.4 Biodegradable mulch films reorganize microbial interaction networks linked to sugarcane productivity

Co-occurrence network analysis based on Spearman correlation showed that BMFs treatments significantly altered rhizosphere bacteria–fungi interaction patterns, manifested as increased network complexity, enhanced functionality of key nodes, and differentiation of interaction types (Figure 4), consistent with previous findings (Liu et al., 2026). The PM network exhibited an isolated interaction pattern dominated by associations between *Ascomycota* and *Proteobacteria*, with sparse intra-bacterial connections (Figures 4a, c), possibly reflecting intense resource competition and weak microbial cooperation under long-term conventional polyethylene mulch, resulting in poor disturbance resistance (Sun et al., 2022). In contrast, BMFs formed denser networks. Compared with PM, node numbers increased by 65.7% and 63.8%, and edge numbers increased by 246.0% and 259.0% under BTK and BTH treatments, respectively, accompanied by reduced modularity, indicating that BMFs markedly enhanced microbial network complexity, connectivity density, and inter-module connections. Meanwhile, compared with PM, the BTH network showed a significant increase in positive links (5.92%), uniquely strengthening the co-occurrence of *Chloroflexi* and *Acidobacteria*. Conversely, the BTK network exhibited the highest proportion of negative correlations (10.37% higher than PM), indicating differentiated regulation of microbial cooperation and competition (Supplementary Table S3). Concurrently, connector taxa under BTK treatment (e.g., *Rhizomicrobium* and *Stenotrophomonas*) linked multiple modules, consistent with their nitrogen-fixation functions (Figure 3h). These results suggest that BTH treatment may promote the formation of more mutualistic interactions, whereas BTK treatment enhances antagonistic or competitive interactions, reflecting differential impacts of mulch degradation products on soil microecological balance. BMFs alleviate microbial resource competition by supplying additional carbon sources, promoting interspecific cooperation and communication and forming more complex interaction networks, consistent with studies showing that exogenous carbon inputs from biodegradable mulches stimulate microbial activity and respiration, thereby enhancing network connectivity (Hao et al., 2024).

Compared with PM, BTH exhibited the highest degree and graph density, indicating strengthened interspecific interactions (*p* < 0.05). Contrastingly, betweenness centralization under BTK was 1.8-fold higher than that of PM, mainly driven by module-bridging taxa such as *Tumebacillus* and *Pedobacter*. Robustness and average path length were comparable among all treatments, indicating that BMFs enhanced network connectivity without compromising overall system stability, and may even improve ecological buffering capacity by increasing network redundancy (Liang et al., 2025). BMFs altered phylogenetic association patterns among microbial taxa, strengthening both inter-domain interactions between fungi and bacteria and intra-domain interactions within bacteria (Li et al., 2025). PM treatment maintained a relatively isolated interaction network centered on *Ascomycota*. In our study, BMFs, particularly BTH, transformed the rhizosphere into a more interconnected microbial system, strengthening cross-domain cooperation and functional redundancy, potentially enhancing sulfur and nitrogen cycling. In contrast, the modular network under PM may restrict resource sharing, consistent with the observed reduction in nutrient-cycling gene abundance (Figures 3g, h).

Changes in microbial interaction patterns were further associated with soil physicochemical properties and sugarcane agronomic traits, and Mantel test results revealed complex, treatment-specific relationships among these factors under different mulch treatments (Figure 5). Among soil factors, pH, SOM, and AK were the core drivers of microbial community differentiation, predicted functional profile variation, and microbial network structure (Figures 3c, d), consistent with previous studies in maize and cotton systems (Hu et al., 2022; Shangguan et al., 2024). BMFs increase SOM through degradation-derived carbon release while regulating rhizosphere pH, directly influencing microbial colonization, metabolism, and interactions, and indirectly shaping microbial communities by altering nutrient availability such as AK. Regarding agronomic trait associations, FUNGuild functions under BTH treatment were significantly positively correlated with sugarcane plant height, possibly related to enrichment of fungal taxa such as *Cochliobolus*, which, although partially pathogenic, may suppress other harmful microbes through resource competition in this study (Louis et al., 2015). By way of contrast, under BTK treatment, graph density was significantly positively correlated with stem diameter, indicating that complex microbial interaction networks shaped by thicker BMFs may promote sugarcane stem development by enhancing nutrient supply efficiency. Under PM treatment, betweenness centralization was significantly negatively correlated with single-stalk weight and yield, further confirming that isolated microbial networks under PE mulch are unfavorable for crop growth. These associations indicate that BMFs optimize soil–microbe–crop interactions by modifying microbial communities, functional genes, and network structures; in particular, BTH treatment increased the number and types of key regulatory taxa and promoted both inter-domain and intra-domain microbial interactions, laying a microecological foundation for improved sugarcane yield and quality.

### 4.5 Biodegradable mulch films promote sugarcane productivity through coordinated soil–microbial responses

BMFs application was associated with improved sugarcane productivity through coordinated changes in rhizosphere environmental properties, microbial community characteristics, and predicted functional potentials. Compared with PM, BMFs significantly increased single stalk weight, millable stalk number, and cane yield by 8.76%−11.68%, 4.99%−7.38%, and 17.11%−17.40%, respectively (*p* < 0.05) (Supplementary Table S1). The cane yield in our trial exceeds the regional predicted average for Yunnan sugarcane areas (90.32 t/ha), which is consistent with the high basal soil fertility of the experimental site and aligns with the finding that soil properties dominate sugarcane yield variation (Zhu et al., 2026). This result also demonstrates that BMF can further unlock yield potential based on conventional fertilization by improving rhizosphere nutrient use efficiency. Moreover, cane yield was positively associated with microbial network complexity (Mantel *r* = 0.72, *p* = 0.001), suggesting that enhanced microbial interactions may contribute to more efficient nutrient transformation processes within the rhizosphere. Previous studies have demonstrated that biodegradable mulching can improve soil microenvironmental conditions and stimulate microbial mediated nutrient transformation (Liang et al., 2025), supporting the cascading effects observed in this study. Our results showed that BMFs increased soil pH and AK, indicating improved rhizosphere nutrient availability and providing favorable conditions for microbial functional enhancement and nutrient cycling. The effects of BMFs on sugarcane performance were thickness dependent. The thinner BTH mulch exhibited stronger microbial community differentiation and greater shifts in predicted functional profiles, whereas the thicker BTK mulch showed relatively stable microbial regulation effects. These differences indicate that mulch thickness represents an important management factor for optimizing microbial-mediated ecological functions under biodegradable mulching systems. Such cascading regulation may be particularly relevant for sugarcane production systems due to their high water demand and long growth duration (Wang F. et al., 2024). Overall, BMFs improved sugarcane growth by integrating soil environmental regulation with microbial ecological processes (Zhang et al., 2024b), providing an environmentally sustainable alternative to conventional polyethylene mulch. Nevertheless, several limitations should be acknowledged. The lack of continuous environmental monitoring and direct characterization of BMF degradation processes limits our understanding of thickness-dependent microbial responses. In addition, yield responses, application costs, degradation byproducts, and long-term effects of BMFs on soil health require further evaluation across different production systems. Future studies integrating long-term field monitoring, degradation dynamics, and multi-omics approaches are needed to improve the understanding and sustainable application of BMFs.

## 5 Conclusion

This study evaluated the effects of biodegradable mulch films (BMFs) with different thicknesses on sugarcane rhizosphere processes and productivity in a field experiment in Yunnan Province, China. Compared with conventional polyethylene mulch (PM), BMFs improved rhizosphere soil conditions by increasing soil pH and available potassium, modifying microbial community composition and assembly processes, and enhancing microbial functional potentials associated with nutrient cycling. These microbial shifts were accompanied by more complex bacteria–fungi interaction networks and improved crop performance, with BMFs increasing cane yield by 17.11–17.40% relative to PM. The effects of BMFs were thickness dependent: the thinner BTH treatment induced stronger microbial community selection and shifts in predicted functional profiles, whereas the thicker BTK treatment exhibited more stable microbial regulation. Overall, BMFs enhanced sugarcane productivity through coordinated regulation of soil properties, the rhizosphere microbiome, and nutrient cycling-related functional potentials, while reducing the risk of plastic residue accumulation, highlighting their potential as sustainable alternatives to conventional polyethylene mulch. Future research should clarify the long-term ecological consequences of BMFs degradation and optimize mulch strategies across diverse agricultural systems.

## Data Availability

The datasets presented in this study can be found in online repositories. The names of the repository/repositories and accession number(s) can be found below: https://www.ncbi.nlm.nih.gov/, PRJNA815949.
