# Supplementary material for: Biodegradable mulch films promote sugarcane growth by regulating rhizosphere microbial communities and predicted nutrient cycling-related functional potentials
Source: Front Microbiol. 2026 Aug 26;17:1918463. doi: 10.3389/fmicb.2026.1918463 (PMC13553762; doi:10.3389/fmicb.2026.1918463)
Supplement: Supplementary file 1 [file Data_Sheet_1.docx]

**Biodegradable mulch films promote sugarcane growth by regulating rhizosphere microbial communities and nutrient cycling**

Qianyuan Duan^1,2#^, Qiang Liu^1,2#^, Xinping Mao^5^, Zhaonian Yuan^4^, Ziqin Pang^3*^, Yufang Shen^1,2*^

^1^ State Key Laboratory of Soil and Water Conservation and Desertification Control, Northwest A&F University, Yangling 712100, China

^2^ College of Natural Resources and Environment, Northwest A&F University, Yangling, 712100, China

^3^ Xianghu Laboratory, Hangzhou, 311231, China

^4^ National Engineering Research Center for Sugarcane, Fujian Agriculture and Forestry University, Fuzhou 350002, China

^5^ Institute of Agricultural Resources and Environment, Ningxia Academy of Agro-forestry Science, Yinchuan, Ningxia Province 750002, China

^#^ These authors contributed equally to this work.

^*^ Corresponding author.

Email: yufangpaper@163.com (Yufang Shen).

Email: ziqintea@126.com (Ziqin Pang).

**Table S1** Agronomic traits and yield of sugarcane under three mulching treatments

| Treatments | PM | BTK | BTH |
| --- | --- | --- | --- |
| Plant Height (cm) | 260.33±8.76a | 270.78±7.57a | 264.44±9.23a |
| Stalk Diameter (cm) | 2.64±0.16a | 2.69±0.11a | 2.83±0.13a |
| Single Stalk Weight (kg) | 1.37±0.04b | 1.53±0.00a | 1.49±0.08ab |
| Brix (°Bx) | 20.42±0.27a | 20.17±0.27a | 20.26±0.25a |
| Sucrose Content (%) | 14.29±0.05a | 14.29±0.04a | 14.27±0.07a |
| Millable Stalks (stalks/mu) | 4971.74±7.07c | 5219.58±13.23b | 5338.97±13.69a |
| Cane Yield (kg/mu) | 6805.50±182.55b | 7989.56±14.60a | 7969.35±440.38a |
| Sugar Yield (kg/mu) | 973.23±28.64b | 1141.99±4.00a | 1137.86±63.78a |

Note: Data are mean ± SE (n = 9). Statistical comparisons were performed by one-way analysis of variance (ANOVA) followed by Fisher’s least significant difference (LSD) multiple-comparison test. Different lowercase letters within a row denote significant differences among treatments at *p* < 0.05. Abbreviations: PM, conventional polyethylene mulch (0.008 mm); BTK, biodegradable mulch films (0.008 mm); BTH, biodegradable mulch films (0.005 mm).

**Table S2** Rhizosphere soil physicochemical properties under three mulching treatments

| Treatments | PM | BTK | BTH |
| --- | --- | --- | --- |
| pH | 4.62±0.05b | 4.75±0.06ab | 4.82±0.06a |
| SOM (g/kg) | 22.03±0.43a | 19.73±1.07a | 21.01±0.83a |
| AN (mg/kg) | 94.11±4.83a | 114.33±11.25a | 102.04±3.05a |
| AP (mg/kg) | 36.22±3.18a | 40.62±5.13a | 29.63±4.67a |
| AK (mg/kg) | 36.67±1.86b | 50.22±4.24a | 52.22±3.13a |

Note: Data are mean ± SE (n = 9) and were compared using one-way ANOVA with Fisher’s LSD post-hoc test. Different lowercase letters within each row indicate significant pairwise differences at *p* < 0.05. Abbreviations: SOM, soil organic matter; AN, available nitrogen; AP, available phosphorus; AK, available potassium; PM, conventional polyethylene mulch (0.008 mm); BTK, biodegradable mulch films (0.008 mm); BTH, biodegradable mulch films (0.005 mm). Methods for soil chemical analyses follow those described in the Methods section.

**Table S3** Network topology metrics of rhizosphere bacterial-fungal OTU co-occurrence networks for different mulching treatments

| Treatments | PM | BTK | BTH |
| --- | --- | --- | --- |
| Positive | 380 | 1152 | 1467 |
| Negative | 74 | 419 | 170 |
| Positive/Sum | 83.70% | 73.33% | 89.62% |
| Negative/Sum | 16.30% | 26.67% | 10.38% |
| Clustering coefficient | 0.439453125 | 0.399678973 | 0.451094891 |
| Heterogeneity | 0.719604989 | 0.998439604 | 0.990789912 |
| Centralization | 0.01624035 | 0.029278483 | 0.036568431 |

Note: Networks were constructed from combined rhizosphere bacterial-fungal OTU datasets (9 replicates per treatment). After initial filtering prior to network analysis, each network contained 1101 OTUs. Significant taxon-taxon associations were identified using Spearman’s rank correlations, with edges retained where |ρ| > 0.6 and *p* < 0.05. Network topology metrics were calculated using the R packages *igraph* (v1.4.3) and *microeco* (v0.14.0). Definitions: Clustering coefficient–a measure of local connectivity, with higher values indicating stronger cohesion among taxa; Heterogeneity–variability in node degrees, with higher values reflecting a more hub-and-periphery structure; Centralization–the extent to which overall connectivity is dominated by a few nodes, with lower values indicating less reliance on individual hubs. Further details on network construction and filtering are provided in Methods.

**Fig.S1** Taxonomic composition and differentially abundant taxa of rhizosphere microbiota under different mulching treatments. (a) Circular hierarchical (cladogram-style) representation of bacterial taxa (OTU level) and their treatment associations. Outer rings indicate taxonomic rank (phylum→genus→OTU); nodes represent taxa and are colored by the treatment in which they are enriched. Plot produced with the R package *circlize* (v0.4.15). (b) Linear discriminant analysis (LDA) score bar plot for fungal taxa identified as differentially abundant among treatments using LEfSe. The x-axis shows LDA scores (log_10_ transformed), indicating effect size; bar color denotes the treatment with enrichment. LEfSe analysis settings are described in Methods. Abbreviations: PM, conventional polyethylene mulch (0.008 mm); BTK, biodegradable mulch films (0.008 mm); BTH, biodegradable mulch films (0.005 mm). Visualization implemented using *ggplot2* (v3.4.4). Sample sizes: n = 9 per treatment.

**Fig.S2** Species contributions to between-group differences in rhizosphere bacterial communities. (a) Bar plots showing the top 20 bacterial species ranked by their average contribution to pairwise community dissimilarities between treatments. The x-axis quantifies each species’ contribution to dissimilarity. (b) Cumulative contribution curves: species are added in descending order of contribution and the total explained dissimilarity is plotted as a function of the number of species included. Contributions were estimated using a Random Forest–based approach to quantify taxa-mediated community variation (see Methods for algorithmic parameters and permutation testing). Statistical notation, abbreviations, and visualization tools as in Fig. S1. Sample size: n = 9 per treatment.

**Fig.S3** Detailed species-level contributions to bacterial community dissimilarity. (a) Dot-and-line plots showing each species’ average contribution (points) and the corresponding cumulative contribution (lines) to pairwise community dissimilarity between treatments. The x-axis quantifies species-mediated community variation. (b) Heatmaps summarizing species’ contribution magnitudes: columns indicate “average contribution” and “cumulative contribution”; color intensity reflects contribution magnitude (darker = larger contribution). Statistical notation, abbreviations, and visualization tools as in Fig. S1. Sample size: n = 9 per treatment.

**Fig.S4** Species contributions to between-group differences in rhizosphere fungal communities. (a) Bar plots of the top 20 fungal species ranked by average contribution to pairwise community dissimilarity between treatments. (b) Cumulative contribution curves showing the progressive explanatory power as taxa are added in descending order of contribution. Contributions were estimated using a Random Forest-based method (see Methods for details). Statistical notation, abbreviations, and visualization tools as in Fig. S1. Sample size: n = 9 per treatment.

**Fig.S5** Detailed species-level contributions to fungal community dissimilarity. (a) Dot-and-line plots: each fungal species’ average contribution (points) and cumulative contribution (curves) to community dissimilarity between treatment pairs. (b) Heatmaps showing contribution magnitude, with the two columns “average contribution” and “cumulative contribution”; darker colors indicate larger contributions. Statistical notation, abbreviations, and visualization tools as in Fig. S1. Sample size: n = 9 per treatment.

**Fig.S6** Analysis of soil carbon and phosphorus cycling, based on KO abundance in the predicted bacterial functions from PICRUSt2. (a) Metabolic pathway distribution of microbial genes involved in carbon cycling under BTH, BTK and PM treatments. Key pathways shown include Embden-Meyerhof-Parnas (glycolysis), Entner-Doudoroff (ED) pathway, Calvin-Benson-Bassham (CBB) cycle, reductive TCA (rTCA) cycle and the Wood-Ljungdahl (WL) pathway. (b) Proportional distribution of genes implicated in phosphorus cycling processes. Key pathways shown transformations of phosphorus-containing metabolites or compounds. Key genetic markers and metabolic pathways reflect microbial mediation of phosphorus dynamics. Circle sizes reflect relative gene abundance, and sectors indicate the proportional contributions of the BTH, BTK, and PM treatments.

**Fig.S7** Mantel test analysis of relationships among soil physicochemical properties, sugarcane core agronomic traits, and rhizosphere microbial indicators under different mulching treatments. (a-c) Relationships between the above variables and microbial functions (PICRUSt2: functional gene prediction; FAPROTAX: bacterial functional annotation; FUNGuild: fungal functional annotation), where (a) corresponds to the BTH treatment, (b) to the BTK treatment, and (c) to the PM treatment. (d-f) Relationships between the above variables and microbial network properties (Robustness, Average path length, Betweenness centralization, and Graph density). Circles represent Mantel test data points; Mantel p-value ranges are distinguished by line colors; Mantel r-value ranges are distinguished by line thickness. Pearson’s r values are accompanied by significance labels: *p* < 0.05 (*) and *p* < 0.01 (**). AN = available nitrogen; AK = available potassium; SOM = soil organic matter; AP = available phosphorus; Brix = sugarcane sugar degree; Single-stalk Weight = sugarcane single-stalk dry weight; Stem Diameter = sugarcane stem diameter; Yield = sugarcane fresh yield; Sugar Yield = sugarcane sugar production; Sugar Content = sugarcane sugar percentage.
